# Plant-based diet for obesity treatment

**DOI:** 10.3389/fnut.2022.952553

**Published:** 2022-09-08

**Authors:** Siti Rohaiza Ahmad

**Affiliations:** Pengiran Anak Puteri Rashidah Sa'adatul Bolkiah (PAPRSB) Institute of Health Sciences, Universiti Brunei Darussalam, Bandar Seri Begawan, Brunei

**Keywords:** plant-based diets, obesity, overweight, weight loss, diabetes, glucose metabolism, RCT

## Abstract

Obesity rates continue to rise, resulting in a global epidemic that shows no sign of slowing down. Our understanding of this complex disease is also constantly evolving, requiring healthcare providers to stay up to date with best practices. The application of plant-based diets (PBDs) may hold the key to a successful weight-control strategy. PBD refers to any dietary pattern that emphasizes the consumption of plant foods while excluding the consumption of most or all animal products. The purpose of this mini-review is to report on the application of PBDs as a potential treatment for obesity. PBDs have also been shown to be beneficial in the treatment of other non-communicable diseases, such as the prevention and treatment of type 2 diabetes. Many of the reported RCTs were of short duration. Longer-term studies, as well as studies focusing on strict adherence to the PBD regime, are needed. PBD is a beneficial approach to improving health, particularly in obese patients. Benefits include weight loss, improved cardiovascular health, lower blood pressure, and improved glucose metabolism.

## Introduction

“Obesity” (Body mass index [BMI] ≥ 30) and “overweight” (BMI 25–29.9) represent abnormal or excessive fat accumulation that may have a negative health impact (1). Obesity is increasing in all age groups. More than 1.9 billion adults (>18 years [y]) are overweight, and 650 million adults are obese, with obesity nearly tripling since 1975 (1, 2). In 2020, 39 million children (<5 y) had a BMI ≥ 25, plus over 340 million children and adolescents (5–19 y) were also obese. The majority of the world's population lives in countries where being overweight or obese kills more people than being underweight (2).

Obesity rates continue to rise, resulting in a global epidemic that shows no signs of slowing down (2). Obesity and related disorders are commonly regarded as problems of developed countries, but they are becoming more prevalent in developing countries as well. Increased BMI is a risk factor for non-communicable diseases, such as diabetes, cardiovascular disease (CVD), and musculoskeletal disorders, resulting in a significant reduction in life quality and expectancy (3). Consequently, there is an urgent need to prevent and control this epidemic. Obesity treatment and management are constantly evolving, as is our understanding of this complex disease. There are challenges for healthcare practitioners to stay up to date with best practices in this fast-moving field (4).

Weight loss success is dependent on optimizing nutrition and physical activity, but also the extent of compliance to treatment. Obesity management is multifaceted, with four key components identified which include diet, activity level, behavioral intervention, and patient management (*via* medication/pharmacotherapy) (4, 5). The purpose of this mini-review is to report the latest published evidence on the efficacy of using plant-based diets (PBDs; defined here as vegan and vegetarian dietary patterns) as a potential treatment for obesity. This review will summarize the current challenges in obesity management and treatment plus discuss dietary management and how PBDs can help with obesity treatment.

### Current management of obesity

Current evidence-based obesity management includes a long-term individual approach by healthcare providers. Fitzpatrick et al. (6) published weight-management counseling guidelines and strategies, as well as a framework for assembling a multidisciplinary team to maximize the patient's weight-management success. The author described the 5As counseling framework for obesity management as follows: Assess, Advise, Agree, Assist, and Arrange. Weight maintenance needs to follow a thorough process that extends from the weight loss phase to the maintenance phase, as demonstrated in a recent systematic review and meta-analysis (8 studies, with a total of 1,454 adult patients) (7). The population of interest consisted of participants who had successfully completed a dietary weight-loss treatment for overweight or obesity and were enrolled in a study using a weight-maintenance intervention (*via* a hypocaloric diet); the outcome of interest was the weight difference between cases and controls after weight-maintenance intervention at fixed follow-up (12 months). The intervention groups, in particular, were given a treatment regimen that included protocols for psychological and motivational intervention, nutritional support, and/or increased physical activity. It was found that weight regain was observed in nearly half of the weight loss studies.

Several factors influence behavioral interventions in achieving weight loss success. It was suggested that interventions should be intense and face-to-face for best results. Chopra et al. (8) identified other factors which impact on the success of behavioral interventions, such as older age, pre-existing heart conditions, and limited fat intake. In addition, patients must strictly follow the lifestyle advice to achieve greater weight loss success. These findings were supported by another study which agreed that high frequency contact with patients in multidisciplinary team interventions led to better weight loss outcomes (9). It was highlighted that additional factors are important including social support, increased disease awareness, motivational interviewing, counseling, goal-setting, and self-monitoring. It was stressed that the intervention should be personalized to meet the needs of the patients. In terms of treatment, it must be ensured that the regime is both safe and effective. Behavioral modification is important in the treatment of obesity; however, achieving and maintaining a healthy weight is difficult. As a result, patients who are overweight should engage in lifestyle interventions as their first line of treatment (10). According to the National Weight Control Registry (NWCR) members, there were a variety of ways for patients to maintain weight loss, which include eating breakfast every day (78%), weighing once weekly (75%), watching less 79 than 10 h of TV per week (62%), and exercising an average of 1 h per day (90%) (11).

### Dietary management of obesity

Dietary management is one method of treatment sought for overweight and obese individuals. Obesity is caused by a long-term energy imbalance between calories consumed and calories used (3). The primary goal of dietary management in overweight and obese individuals is to create an energy deficit that results in weight loss and can be sustained over time (3, 12).

Individuals with metabolic syndrome (MetS) may have some of the sequelae associated with elevated BMI but do not have that specific finding; however, they may have hypertension, dyslipidemia, and other conditions. They are at increased risk for developing heart disease, diabetes, and other serious health issues (13). Understanding that individuals with obesity are phenotypically heterogeneous (i.e., in terms of body composition phenotypes, such as body fat and lean mass, plus metabolic, and functional variables) is a concept that has been defined in the scientific literature (14, 15). Pujia et al. (16) stated that BMI is only as a guidance for classifying obesity, and a new approach that includes assessment of body composition and metabolic parameters (such as biomarkers) is suggested. This approach results in personalized therapies that may help to reduce the risk of non-communicable diseases in the most effective way. Precision nutrition has the potential to offer more effective approaches tailored to individual characteristics, such as the genome, metabolome, and microbiome, taking personalized disease management and prevention one step further (17). This area of research, however, is still in its early stages and is not yet ready for widespread clinical use.

Numerous diets have been proposed as therapeutic strategies for obesity. Dietary strategies such as Paleolithic, ketogenic, Mediterranean, high-protein, plant-based, low-carbohydrate, and intermittent fasting have grown in popularity due to their alleged benefits for weight loss and metabolic disease (18). Over the last two decades, various weight-control strategies have been introduced (19). Unfortunately, these approaches have had little impact. At the same time, numerous publications suggest that plants may hold the key to a successful weight-control strategy (19–21). There is a large body of evidence supporting the benefits of PBDs for obesity and chronic disease prevention from prospective cohort studies and controlled trials (22). PBDs appear to be a risk-free, sensible, and long-term remedy to the obesity crisis (20, 23). The purpose of this mini-review is to report the latest published evidence on the efficacy of using PBDs as a potential treatment for obesity.

## What are PBDs?

Plant-based diet refers to any dietary pattern that emphasizes the consumption of plant-based foods while excluding most or all animal products (22). PBD include both vegan and lacto-ovo-vegetarian diets and are becoming more popular in the Western world for a variety of reasons, such as concerns about human and environmental health (24–27). PBDs may be a personal dietary preference for some individuals, but depending on their composition, they can be health-promoting or non-health promoting (17). Healthy PBD includes unprocessed plant foods, such as fruits, vegetables, whole grains, legumes, nuts, and seeds, whereas harmful PBD includes refined grains and more sugars (20, 22).

Recent food-based dietary guidelines (FBDG) advise consuming more plant-based foods and minimizing animal-based foods to achieve environmental sustainability (28, 29). PBDs are more environmentally sustainable than meat-based diets and have a lower environmental impact, such as lower levels of greenhouse gas emissions (24, 25, 30). There is consistent evidence that a diet rich in plant-based foods and low in animal foods (particularly red meat), is healthier and has a lower environmental impact, as well as lower in total energy (31). PBDs have been shown to be micronutrient dense, but less energy-dense (32). The only nutrient found to be lacking when completely eliminating animal products is vitamin B12 that may require supplementation (33). Another nutrient that may need to be taken into consideration is calcium, but supplementation is not advisable because of its harmful effects, i.e., increased risk of CVDs (34). Interestingly, a study demonstrated that, despite the calcium level dropping at 3 weeks, it appears to rise after 6 weeks of adhering to a healthy PBD (33). Vegan diets have been linked to better gut microbiota symbiosis, enhanced insulin sensitivity, peroxisome proliferator-activated receptors (PPARs) stimulation, and upregulation of mitochondrial uncoupling proteins (19).

### Plant-based diet for weight loss–Some theories

In the literature, there are several theories about why PBD is suitable for weight loss. The most widely accepted theory is that plants have low-calorie density (the number of kilocalories (kcal) per unit weight of food) and reduced fat content (19). Low-calorie-dense foods are more beneficial for weight loss than smaller portion sizes (19). In addition, some calories are trapped within indigestible cell walls when obtaining macronutrients from structurally intact plant foods, which reduce the bioavailable food energy compared to the results of “available” energy measured with a bomb calorimeter. Some experimental evidence supports the influence of PBD on gut microbiota (35). A recent RCT found that a low-fat vegan diet increased *Faecalibacterium prausnitzii* and decreased *Bacteroides fragilis*, which was associated with a greater loss of body weight, fat mass, visceral fat, and an increase in insulin sensitivity (36).

Several compounds found in plants have been identified as potentially beneficial to weight loss (37, 38). These anti-obesity compounds include polyphenols, phenolic acids, flavonoids, and alkaloids. Consumption of a diet high in active anti-obesity natural compounds is a promising strategy for suppressing lipid accumulation and adipogenesis (37). Curcumin is another compound found in the rhizome of *Curcuma longa L*., one of the many plants that may act as an anti-obesity agent (39). The key components of PBD are lower calorie density and lower fat intake. The combination of these two factors is the likely rationale for the effectiveness of this weight-control approach (19).

### Plant-based diet for weight loss and other health parameters–Recent evidence

The implementation of PBD among overweight participants resulted in improvements in body composition, weight loss, and insulin resistance according to an RCT by Kahleova et al. (40). A 16-week RCT using low-fat PBD for overweight adults resulted in improved beta-cell function and insulin sensitivity (41)–see Table 1. These findings imply that PBD could be useful in the treatment of diabetes, one of the health risks associated with being obese or overweight (51). Another 16-week RCT with a low-fat vegan diet among overweight people reported that it reduced body weight by lowering energy intake and increasing postprandial diet-induced thermogenesis (improves insulin sensitivity) (36).

**Table 1:** Selected studies on the metabolic/health outcome of plant-based diets (PBDs)– among overweight/obese patients.

**References**	**Study participant/ numbers**	**Type of study**	**Type of PBDs used in the study**	**Outcome**
Kahleova et al. (40)	Overweight participants (*n* = 75)	Randomized-a plant-based diet	Vegan: low-fat vegan diet consisting of vegetables, grains, legumes, and fruits. No animal products and added oils.	Improvements in body composition and reductions in both body weight and insulin resistance (body weight, fat mass, and insulin resistance markers)
Kahleova et al. (41)	Overweight adults (*n* = 38)	16-Week Randomized Clinical Trial- low-fat plant-based diet	Low-fat vegan diet consisting of vegetables, grains, legumes, and fruits. No animal products and added fats.	Beta-cell function and insulin sensitivity were significantly improved
Kahleova et al. (36)	The intervention group – Overweight adults (*n* = 122)	16-Week Randomized Clinical Trial-	Low-fat vegan diet-vegetables, grains, legumes, and fruits without animal products or added fats.	A low-fat plant-based diet reduces body weight by lowering energy intake and increasing postprandial metabolism. The modifications are linked to decreased hepatocellular and intramyocellular fat and risen insulin sensitivity.
Klementova et al. (42)	Three groups: Obese, Type 2 diabetes and Healthy adults (*n* = 60)	A randomized crossover design	A single plant-based tofu burger (Vegetarian-meal)	Improve in the section of gastrointestinal hormones and satiety
Johannesen et al. (43)	Systematic Review	RCTs investigating the effect of a plant-based dietary intervention on outcomes related to glucose metabolism in human subjects compared to an omnivorous diet	PBDs vs. omnivorous diet	A transition to a plant-based diet may improve glycemic control in individuals with type 2 diabetes and/or obesity. However, the data were somewhat contradictory, and the included trials reported findings based on different intervention diets and study populations. Overall, the current findings do not allow for clear conclusions about the effects of various plant-based diets.
Zhu et al. (44)	710 participants (aged 26–70 years) with overweight or obesity and pre-diabetes	3-year weight-loss maintenance phase of the PREVIEW intervention was analyzed.	Impact of specific plant foods on health	Long-term consumption of nuts, fruits, and vegetables may be beneficial for weight management and cardiometabolic health, whereas overall PBDs may improve weight management only
Kahleova et al. (45)	Overweight participants (*n* = 75) were randomized to follow a low-fat vegan (*n* = 38) or control diet (*n* = 37) for 16 weeks.	16-Week RCTs- low-fat vegan diet or control diet	Low-fat vegan diet consisting of vegetables, grains, legumes, and fruits. They were also asked to avoid added oils.	Reduced intake of saturated and trans fats, as well as an increase in the relative content of polyunsaturated fatty acids, particularly linoleic and -linolenic acids, are linked to lower fat mass, insulin resistance, and increased insulin secretion.
Remde et al. (46)		Systematic Review	Plant-predominant (vegan, vegetarian, plant-based whole foods)	Improved weight control and cardiometabolic outcomes
Argyridou et al. (47)	23 regular meat eaters with dysglycemia or obesity	Interventional single-group prospective trial involved	Vegan diet from Week 1 to Week 8. Followed by 4-week period of unrestricted diet	TMAO levels (marginal mean) were reduced after weeks 1 and 8 of a vegan diet compared to baseline. Levels rebounded at week 12, after the unrestricted diet.
Garousi et al. (48)	75 overweight/obese adults with non-alcoholic fatty acid liver disease (NAFLD)	RCT	Lacto-ovo-vegetarian diet	NAFLD improvement, anthropometric measures, glycaemic-related markers, and lipid profiles
Sofi et al. (49)	118 overweight omnivores	RCT	Lacto-ovo vegetarian diet + individual counseling sessions	Reductions in total body weight, BMI, and total fat mass
Huang et al. (50)	A total of 1,151 subjects who received the intervention over a median duration of 18 weeks	Meta-analysis of RCT	Vegan, lacto-ovo-vegetarian and habitual diet were compared.	Vegetarian diets lost more weight than those assigned to control diets. But vegan diets lost more weight than those randomized to lacto-ovo-vegetarian diets

An interventional single-group prospective trial demonstrated that trimethylamine N-oxide (TMAO–a reliable indicator of CVD risks) levels were lower after 1 and 8 weeks on a vegan diet compared to baseline (47). Therefore, vegan diet maybe an effective strategy for reducing plasma TMAO in individuals with dysglycemia or obesity. Additionally, in another short-term 8-week RCTs on PBD, participants achieved weight loss, blood pressure reduction, plus a reduction in low-density lipoprotein (LDL), and cholesterol levels (52). A recent systematic review by Remde et al. (46), demonstrated that PBD, in general, demonstrated improved weight control and cardiometabolic outcomes. Healthy plant-based foods play a beneficial role in lowering cardiovascular mortality and CVD according to the findings of a meta-analysis and systematic review of prospect cohort studies (53). Interestingly, another study reported that, PBD (which included Mediterranean, vegetarian, and vegan diets) improved TMAO levels, whereas animal-based diets appear to have the contrary effect (54). However, long-term data on compliance to PBD and weight regain, as well as cardiometabolic risk factors during weight-loss maintenance, are still largely lacking.

Controlling satiety (to prevent overnutrition) is another treatment and management goal for overweight/obese people. Interestingly, Klementova et al. (42) demonstrated that a single PBD exposure (PBD with tofu) resulted in an increase in gastrointestinal hormones for glucose metabolism and satiety. Therefore, plant foods, just like some medication, can also be useful to control satiety. PBD may be beneficial in the prevention of type 2 diabetes, a common comorbidity among obese individuals, because of the ability of plant foods to ameliorate glucose metabolism. Diet transition to a PBD may improve glycemic control in individuals with type 2 diabetes and/or obesity according to a systematic review published in 2020 by Johannesen et al. However, the current data are somewhat contradictory because the trials were based on different diets and study populations. The current studies do not allow for definitive conclusions about the effects of different PBDs.

Zhu et al. (44) conducted the first long-term PBD study, which included 710 participants in a 3-y weight loss maintenance program. The study reported that the long-term consumption of nuts, fruits, and vegetables may benefit weight management and cardiac health, whereas an all-plant diet may only improve weight management. Interestingly, in another study, reduced consumption of saturated and trans fats, as well as an increase in the relative content of polyunsaturated fatty acids, particularly linoleic and linolenic acids, was associated with lower fat mass, insulin resistance, and increased insulin secretion (45). In Slovenia, participants who voluntarily participated in the whole-food, plant-based (WFPB) lifestyle (whole or minimally processed plant foods) for any length of time (0.5–10 y) had normal BMI and body fat values, were physically very active, had good sleep quality, and had low levels of perceived stress (55).

The previous studies on vegetarian diets, in addition to vegan diets, appear to have a positive impact on obesity management as well. Participants who followed a lacto-ovo vegetarian diet improved their non-alcoholic fatty acid liver disease (NAFLD), anthropometric measurements, blood glucose levels, and lipid profiles according to Garousi et al. (48). Similarly, another RCT reported that a lacto-ovo vegetarian diet combined with individual counseling sessions resulted in lower weight, BMI, and total fat mass (49). A meta-analysis of 1,151 patients found that vegan diets resulted in greater weight loss than lacto-ovo-vegetarian diets (50).

It is critical for overweight/obesity nutritional management to provide not only a reduction in body mass, but also a better overall health status, such as lower cholesterol levels, lower triglyceride levels, and better glycemic control (19).

## Conclusion

In conclusion, PBDs have been shown to be effective in the treatment of obesity. PBDs also benefit the treatment, prevention, and reversal of other illnesses, such as type 2 diabetes and hypertension (56–58). Several of the RCTs discussed here were of short duration, typically 16 weeks, with only one study lasting up to 3 y (44). More long-term studies are required, as well as studies on strict adherence to the PBD regime (19, 46). Research into the weight loss effects of PBD with more diverse populations, such as older adults, is also required (23). Therefore, more long-term studies and RCTs are needed to investigate the role of PBD in the treatment of obesity and other comorbidities.

In conclusion, PBDs are beneficial for improving health, especially in obese patients, by helping to achieve weight loss, improved cardiovascular health, lower blood pressure, and improved glucose metabolism.

## Author contributions

SRA wrote the manuscript.

## Funding

This work was funded by Universiti Brunei Darussalam (PAPRSBIHS UBD Allied Grant) (No. UBD/RSCH/1.6/FICBF(a)/2022/026).

## Conflict of interest

The author declares that the research was conducted in the absence of any commercial or financial relationships that could be construed as a potential conflict of interest.

## Publisher's note

All claims expressed in this article are solely those of the authors and do not necessarily represent those of their affiliated organizations, or those of the publisher, the editors and the reviewers. Any product that may be evaluated in this article, or claim that may be made by its manufacturer, is not guaranteed or endorsed by the publisher.
